# Apigenin Increases Natural Killer Cytotoxicity to Human *Hepatocellular Carcinoma* Expressing HIF-1α through High Interaction of CD95/CD95L

**DOI:** 10.4014/jmb.2201.01010

**Published:** 2022-02-27

**Authors:** Hwan Hee Lee, Hyosun Cho

**Affiliations:** 1 Department of Pharmacy, Duksung Women’s University, Seoul 01369, Republic of Korea; 2 Duksung Innovative Drug Center, Duksung Women’s University, Seoul 01369, Republic of Korea

**Keywords:** Apigenin, NK, HCC, HIF-1α, CD95L(FasL;CD178)

## Abstract

Natural killer (NK) cell activity is more attenuated in hepatocellular carcinoma (HCC) patients than normal. Hypoxic-inducible factor (HIF)-1α is highly expressed in tumors to maintain their metabolism in a hypoxic environment. The expression of HIF-1α in cancers can lead to cell growth, proliferation, invasion/metastasis and immune escape. Although apigenin, a flavonoid, is known to have various biological activities, it has not been demonstrated in NK cell immune activity in HCC cells. In this study, NK-92 cells were directly cocultured with HCC SK-Hep1 cells for 24 h to evaluate NK cell activity in HCC cells or HCC cells expressing HIF-1α by apigenin. NK cell cytotoxicity to HCC cells expressing HIF-1α was significantly increased, and NK cell-activating receptors, NKG2D, NKp30 and NKp44 were highly expressed. The activating effect of apigenin on NK cells substantially induced apoptosis in HCC cells expressing HIF-1α through high expression of CD95L on the surface of NK-92 cells. Moreover, apigenin excellently inhibited the level of TGF-β1 in a coculture of NK cells and HCC cells. In conclusion, apigenin seems to be a good compound that increases NK cell cytotoxicity to HCC cells by controlling HIF-1α expression.

## Introduction

Hepatocellular carcinoma (HCC) is a common malignancy that ranks fifth in incidence and third in cancer-related mortality worldwide [1]. HCC can be caused by many factors such as infection with hepatitis B or C virus, alcoholism, and diabetes, which are closely related to chronic inflammation [2]. Most patients who have HCC also suffer from diseases such as cirrhosis that can hinder treatment of HCC due to its potential for developing an unpredictable pathogenetic mechanism [3, 4]. Hypoxia-inducible factor (HIF)-1α is commonly expressed in many cancers because of the hypoxic environment that the cancer metabolic system induces [5]. The expression of HIF-1α in cancer results in gene stability, which leads to the promotion of cell growth, proliferation, invasion/metastasis and metabolic adaptation in a hypoxic condition [6, 7]. This was also recently reported to disturb the effective immune actions of T and NK or NKT cells in cancer [8].

Natural killer (NK) cells are innate lymphocytes that have the ability to kill cancer or stressed cells by producing cytotoxic proteins such as granzyme and perforin [9] while also producing various cytokines that can help adaptive immune cells. In the human liver, NK and NKT cells are present at a high frequency (about 55%) and are important as the first line of defense of the liver against pathogens. Therefore, in the development of HCC, NK cells play a very important role in immune surveillance. However, several studies have revealed that a low number of NK cells is recruited into tumors [10] and in low cytotoxicity to tumor cells [11]. The function of NK cells is controlled by their surface proteins, and in particular, the balance of NK activating and inhibitory receptors decides NK cell activity against targets. Activating NK cells produce cytolytic proteins such as granzymes and perforin to kill target cells. The granzyme B (GrzB) released from NK cells stimulates death receptors on transformed or viral-infected cells and initiates caspase cascades [12]. NK activity is regulated by various surface proteins including NK-activating receptors such as natural-killer group 2, member D (NKG2D) and natural cytotoxicity receptors (NKp30 and NKp44) in humans [13] and death ligands such as CD95L [12]. In cellular cytotoxicity, CD95L on the surface of NK cells can activate the death receptor CD95 on the surface of target cells, which induces apoptosis and starts a cascade of caspase-8 [14].

Apigenin, which belongs to a class of flavonoids, is found in various plants including parsley, grape, apple and chamomile and has various biological activities including antioxidant, anti-inflammatory, antibacterial, antiviral, and anticancer effects [15-19]. Moreover, recent studies showed that it possesses the potential to play a role as an immunomodulator of NK cells through an increase in NK proliferation by apigenin [20]. The anticancer or antimetastatic effects of apigenin in various cancers have been reported in many studies, but its effect on NK activity in cancers is not yet proved. Therefore, we tried to evaluate the effect of apigenin on NK activity in a coculture of HCC cells or HCC cells expressing HIF-1α. In this study, we found that apigenin enhanced NK cytotoxicity to HCC cells through high expression of CD95L on the surface of NK cells.

## Materials and Methods

### Specimen Preparation

Apigenin (≥95% pure) and cobalt chloride (CoCl_2_) were purchased from Sigma-Aldrich, Inc. (USA). Apigenin and CoCl_2_ were dissolved in sterile water, diluted in complete media and filtered to a 0.2 μm pore size. To induce the expression of HIF-1α in cells, CoCl_2_ was used at a concentration of 250 μM.

### Cell Lines and Culture

Both NK cell line NK-92 and HCC cell line SK-Hep1 were obtained from American Type Culture Collection (ATCC, USA). NK-92 cells were cultured in alpha-MEM (Gibco, USA) with the addition of 20% fetal bovine serum (FBS; Gibco), 100 U/ml penicillin and streptomycin (Gibco), 0.1 mM 2-mercaptoethanol (Sigma Aldrich), and rhIL-2 (200 U/ml, BioLegend, USA). SK-Hep1 cells were cultured in Dulbecco’s Modified Eagle Medium (DMEM; Gibco) with 10% FBS (Gibco) and 100 U/ml penicillin and streptomycin (Gibco). All were maintained at 37°C in a humidified atmosphere with 5% CO2.

### NK Cell Proliferation Using CCK-8 Assay

The NK cell viability on apigenin was examined by Cell Counting Kit (CCK)-8 assay (Dojindo, Japan). Briefly, NK cells were plated at a density of 5 × 10^4^ cells/well with a various concentrations of apigenin (0, 12.5, 25, 50, 100 and 200 μM) in a 96-well flat bottom plate and incubated for 24 h. After incubation, the cells were treated with CCK-8 solution for 3 h and then measured at the absorbance of 450 nm using a microplate reader.

### NK Cytotoxic Effect on Target Cells by LDH-Release Assay

The NK-92 cytotoxic effect on target cells was identified by a CytoTox96 Non-Radioactive Cytotoxicity Assay Kit (Promega, USA). Briefly, target (SK-Hep1) cells were plated at 5 × 10^3^ cells each in a 96-well flat bottom plate, incubated overnight for 18-24 h and then cocultured with NK-92 cells with or without apigenin treatment (50 μM) for 24 h. A concentration of apigenin was used as previously described [20]. After 24 h, the supernatants were transferred to each well of a fresh 96-well plate, incubated in CytoTox96 reagent for 30 min, and then stop solution was added. The absorbance was assessed at 490 nm within 1 h using a microplate reader.

### Surface Proteins of NK Cells Cocultured with Target Cells Using Flow Cytometry

Surface proteins of NK cells were identified using flow cytometry analysis. Briefly, target cells were plated at a density of 5×10^5^ cells in a 6-well plate and incubated overnight. After incubation, the cells were cocultured with NK-92 cells at 1 × 10^6^ cells for 24 h. The cells were then harvested, stained with anti-CD178-PE, anti-NKG2D-PE, anti-NKp30-PE, anti-CD56-APC, and VP for 30 min, and detected by flow cytometry (Novocyte Flow Cytometer, ACEA Biosciences, USA). All fluorescence antibodies were purchased from BD Biosciences Inc. The positive population of NK cells was analyzed within a range of anti-CD56-APC, and the value to each antibody was compensated by comparison of each emission.

### Western Blot Analysis

Protein expression was examined using Western blot analysis. All protein specimens were extracted from cells cocultured by protein extraction buffer (Intron, Korea). The extracted proteins were quantified using the Bradford (Coomassie blue) assay (Gendepot, USA) and then separated by electrophoresis, transferred to polyvinylidene fluoride (PVFD) microporous membranes (Millipore, USA), and blotted with first and second antibodies. The membranes were soaked in an enhanced chemiluminescent detection solution and then visualized under Chemi-doc (Millipore). First antibodies: anti-HIF-1α (Cell Signaling, USA), granzyme-B (Santa Cruz Biotechnology, USA), anti-caspase (or cleaved caspase)-3,7,8,9 (Cell signaling), anti-Smac (Santa Cruz Biotechnology), anti-XIAP (Santa Cruz Biotechnology), anti-FADD (Santa Cruz Biotechnology), anti-Fas (Santa Cruz Biotechnology), and anti-GAPDH (Santa Cruz Biotechnology).

### ELISA Assay

The quantitation of cytokines was examined by BD OptEIA Set Human IFN-γ, TGF-β1, IL-10 (BD Bioscience) and Human ELISA Kit IL-18 and IL-21 (Invitrogen, USA). Briefly, target cells were cultured in a 6-well plate overnight and cocultured with effector cells (NK-92, ratio of E:T = 2:1) for 24 h with or without apigenin treatment (50 μM). After the coculture, the supernatants were harvested for ELISA assay. All experiments were conducted according to the manufacturer’s instructions. The absorbance of IFN-γ, TGF-β1 and IL-10 was measured at 450 nm with λ correction at 570 nm, and the absorbance of IL-18 and IL-21 was assessed at 450 nm with λ correction at 620 nm using a microplate reader (BMG Labtech, Germany).

### Statistical Analyses

All data were analyzed using Microsoft Excel. The results were presented as means ± SD, and the comparison of several means was performed by one-way or two-way analysis of variance followed by Fisher’s exact test. Differences between groups at a *p*-value of less than 0.05 were considered significant.

## Results

### Increase in NK Cytotoxicity through High Secretion of Granzyme B from NK Cells to HIF-1α-Expressing HCC Cells by Apigenin

**Fig. 1:**
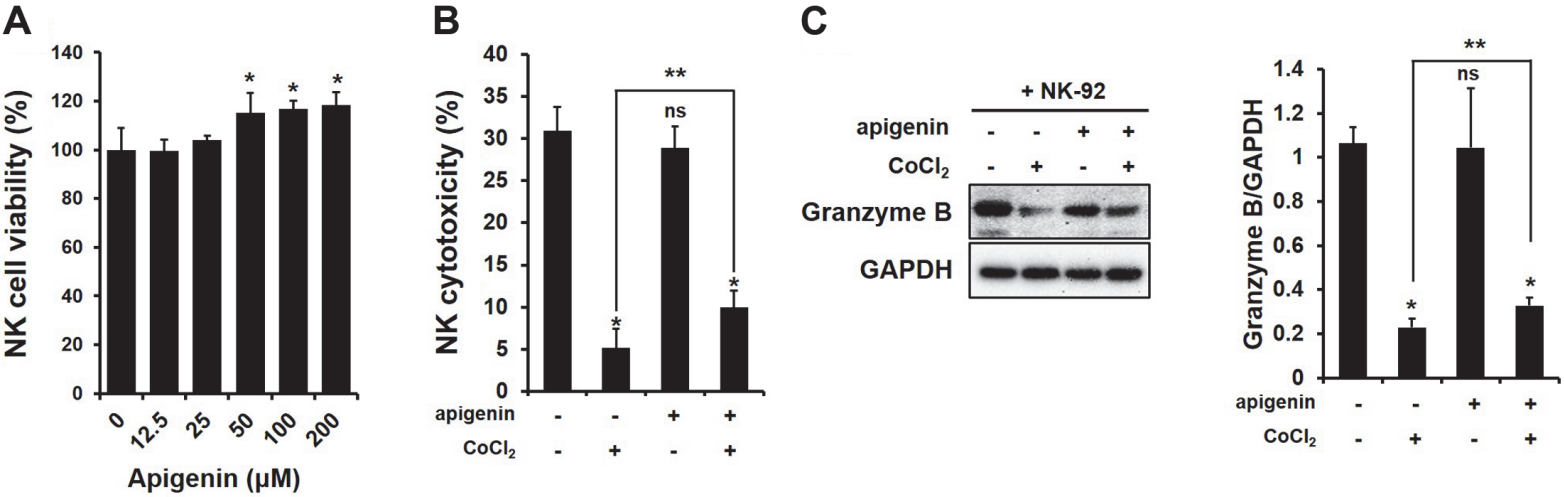
Increase in NK cytotoxicity through high secretion of granzyme (Grz)-B from NK cell to target cell. (**A**) NK cell viability on various concentration of apigenin using CCK-8 assay. (**B**) cytotoxicity and (**C**) granzyme B secreted from NK cells cocultured with HCC cells with or without treatment with CoCl_2_ (250 μM) and apigenin (50 μM). All data were presented as means ± SD from three independent experiments. *p < 0.05 vs non-treated; **p < 0.05 vs CoCl_2_.

To identify the increase in NK cytotoxicity to HCC cells by apigenin by LDH-release assay, a secretion of perforin and granzyme from NK cells was examined in a coculture of HCC cells with or without apigenin treatment (50 μM). The concentration of apigenin used in NK cytotoxicity to HCC cells was examined by CCK-8 assay. As shown in Fig. 1A, apigenin did not have a negative effect on NK cell viability while seeming to increase cell proliferation at over 50 μM (*p < 0.05). NK cytotoxicity to target cells showed no change with apigenin treatment (Fig. 1B; control; 30.96%, apigenin; 28.87%, ns). The secretion of GrzB from NK cells to target cells also showed no significant difference between control and apigenin treatment (Fig. 1C, ns). However, apigenin interestingly increased NK cytotoxicity and the production of GrzB in a coculture of NK and HCC cells when HIF-1α was expressed (NK cytotoxicity; Fig. 1B; CoCl_2_ 4.68%, CoCl_2_+apigenin 9.99%; *,**p < 0.05)

### Increased Expression of NK-Activating Receptors NKG2D, NKp30 and NKp44 on the Surface of NK Cells in Coculture of HIF-1α-Expressing HCC by Apigenin

**Fig. 2:**
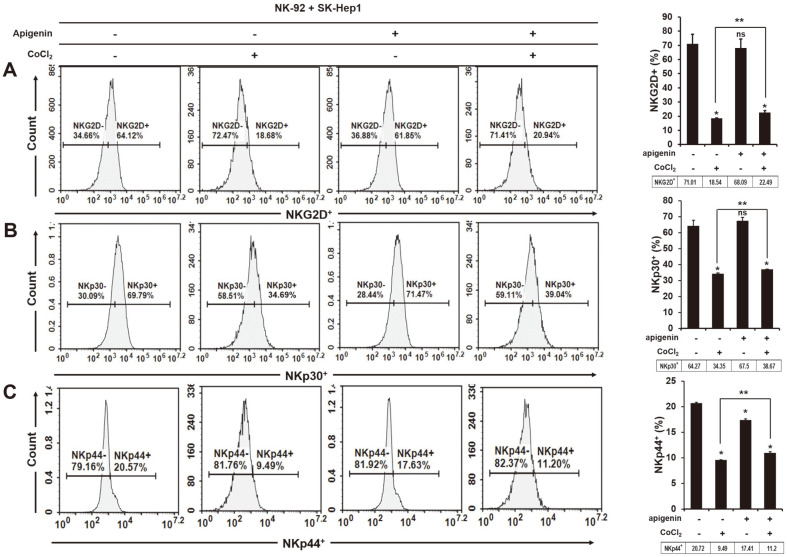
Increased expression of human NK-activating receptors, NKG2D, NKp30 and NKp44 on the surface of NK cells cocultured with HIF-1α-expressing HCC cells by apigenin treatment. The frequency of (**A**) NKG2D, (**B**) NKp30, and (**C**) NKp44 on the surface of NK cells cocultured with HCC cells in the presence or absence of CoCl_2_ (250 μM) and apigenin (50 μM). *p < 0.05 vs non-treated; **p < 0.05 vs CoCl_2_.

NK cell activity depends on a balance between NK-activating and inhibitory receptors on the cell surface. Thus, we identified the expression of human NK-activating receptors on the surface of NK-92 cells cocultured with HCC with or without treatment with CoCl_2_ (250 μM) and apigenin (50 μM). NK-activating receptors NKG2D, NKp30 and NKp44 were examined by staining of fluorescence antibodies (anti-NKG2D-APC, anti-NKp30-PE, and anti-NKp44-PE) using flow cytometry. In Figs. 2A-2C, NKG2D and NKp30 were similarly expressed in NK-92 cells cocultured with HCC and apigenin-treated HCC cells (NKG2D+; non-treated 71.01%, treated 68.09%; NKp30^+^; non-treated 64.27%, treated 67.50%; ns); however, the expression of NK44 on NK-92 was significantly lower in a coculture of HCC treated with apigenin (NKp44+; non-treated 20.72%, treated 17.41%, *p < 0.05). Apigenin had no effect on NK cell activity against HCC cells, but interestingly, it activated NK cells toward HCC cells when HIF-1α was expressed. The expression of NKG2D, NKp30 and NKp44 was higher in a coculture of NK cells and HIF-1α-expressing HCC cells with apigenin treatment than without (Figs. 2A-2C, NKG2D^+^; CoCl_2_ 18.54%, CoCl_2_+apigenin 24.49%, NKp30^+^; CoCl_2_ 34.35%, CoCl_2_+apigenin 38.67%, NKp44^+^; CoCl_2_ 9.49%, CoCl_2_+apigenin 11.2%, **p < 0.05).

### Decreased Expression of HIF-1α in HCC Cells Cocultured with NK Cells by Apigenin

**Fig. 3:**
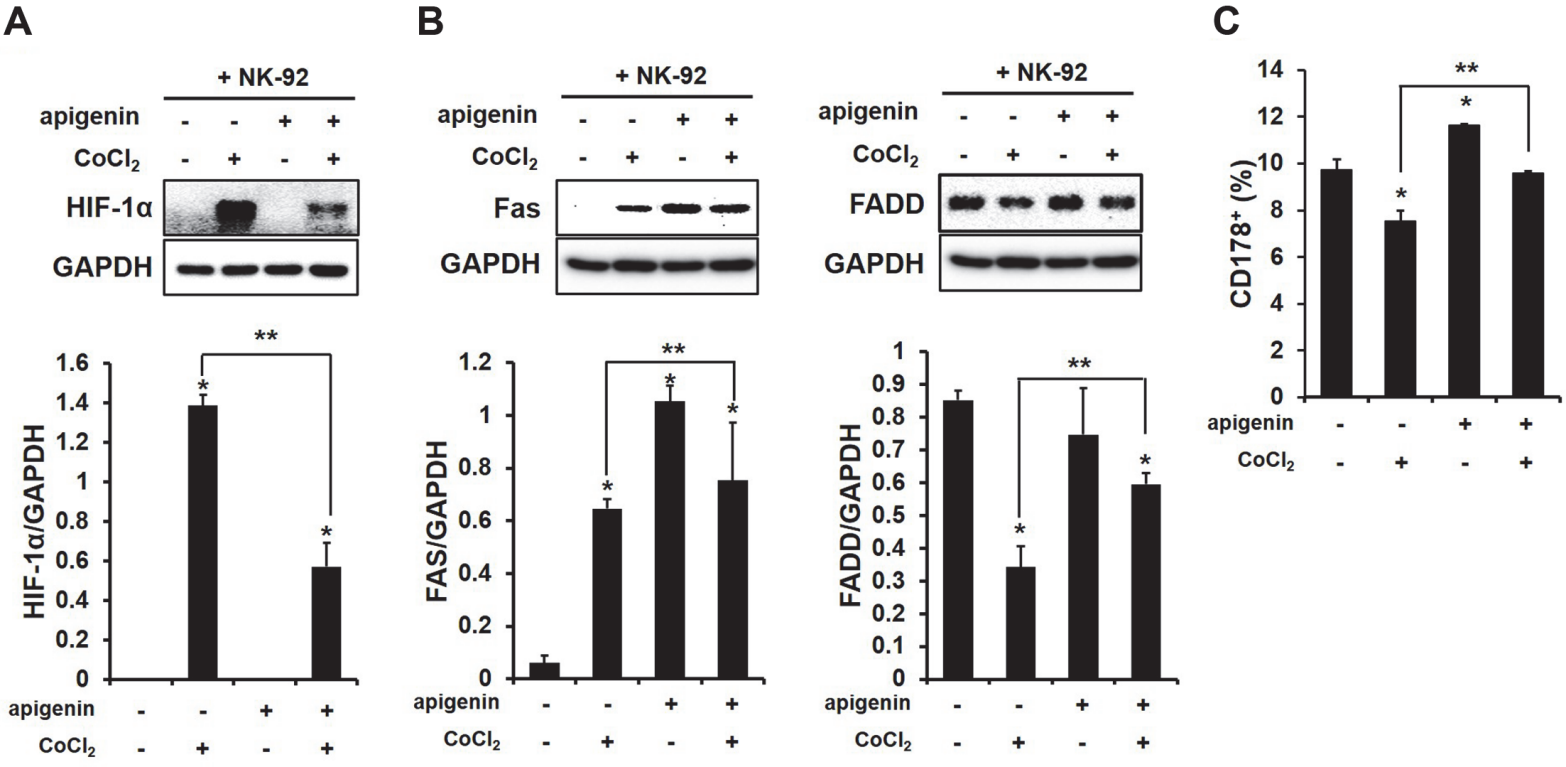
High interaction of Fas (CD95)/FasL (CD95L;CD178) between NK cells and HCC cells through the inhibitory effect of apigenin in HIF-1α expression. The expression of (**A**) HIF-1α, (**B**) Fas and FADD in HCC cells cocultured with NK cells with or without apigenin treatment (50 μM) using Western blot analysis, and (**C**) the positive population of CD178 on the surface of NK cells cocultured with HCC cells by flow cytometry analysis. Data were compared at *p < 0.05 vs non-treated and **p < 0.05 vs CoCl_2_.

To recognize whether apigenin inhibits HIF-1α expression, we investigated the expression of HIF-1α in HCC cells cocultured with NK cells using Western blot analysis. As shown in Fig. 3A, the expression of HIF-1α in HCC cells cocultured with NK cells was significantly lowered when treated with apigenin (*,**p < 0.05).

### Higher Interaction of Fas (CD95)/FasL (CD95L; CD178) between NK Cells and HCC Cells by Apigenin

The secretion of GrzB from NK cells to HCC cells was significantly increased by apigenin (Fig. 1C). According to recent reports, GrzB stimulates a death receptor on the surface of target cells and subsequently induces the apoptosis of target cells [12]. Thus, we tried to examine a death receptor associated with GrzB. In Figs. 3B and 3C, the expression of CD95 (Fas) significantly increased after treatment with apigenin, and FADD, which is an adaptor protein of the bridge Fas receptor was also highly increased with apigenin treatment. Moreover, CD178 (CD95L) on the surface of NK-92 cells was significantly upregulated by apigenin regardless of HIF-1α expression (Fig. 3C, CD178^+^; CoCl_2_ 8.1%, apigenin 11.72%, CoCl_2_+apigenin 9.69%, *,**p < 0.05).

### Increased CD95-Mediated Apoptosis in HCC Expressing HIF-1α by Expressing High CD95L (CD178) on the Surface of NK Cells by Apigenin

**Fig. 4:**
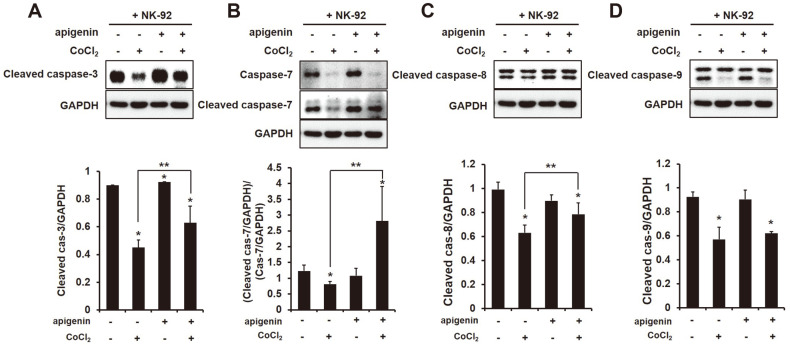
High induction of apoptosis in HCC cells cocultured with NK cells by apigenin. The expression of (**A**) cleaved caspase-3, (**B**) (cleaved or) caspase-7, (**C**) cleaved caspase-8, (**D**) cleaved caspase-9 in HCC cells cocultured with NK cells treated or not treated with CoCl_2_ (250 μM) and apigenin (50 μM). All data were compared at *p < 0.05 vs non-treated and **p < 0.05 vs CoCl_2_, and presented as means ± SD from three independent experiments.

The receptor CD95 binds to CD95L which is implicated in immune homeostasis and immune surveillance and is expressed on the surface of activated T lymphocytes and NK cells [21, 22]. Binding CD95L to CD95 stimulates the activation of FADD, and that triggers apoptosis by the activation of caspase-8. CD95L (CD178) on the surface of NK-92 cells and CD95 (Fas) and FADD in HCC cells increased with apigenin treatment (Fig. 3C). Thus, to verify whether apigenin actually activates the caspases in HCC cells cocultured with NK-92 cells, the expression of caspases (-3,-7,-8 and -9) was investigated by Western blot analysis. The expression of cleaved caspase-8 showed no difference in HCC cells cocultured with NK-92 cells by apigenin but was significant when HIF-1α was expressed (Fig. 4C). Moreover, as shown in Figs. 4A and 4B, the effectors caspase-3 and caspase-7 were significantly activated in HCC expressing HIF-1α cocultured with NK-92 cells by apigenin. The expression of cleaved caspase-9, however, showed no difference between HCC cells in the presence or absence of apigenin (Fig. 4D).

### Decrease in TGF-β1 Released from NK-92 Cells Cocultured with HCC Cells Regardless of HIF-1α Expression by Apigenin

**Fig. 5:**
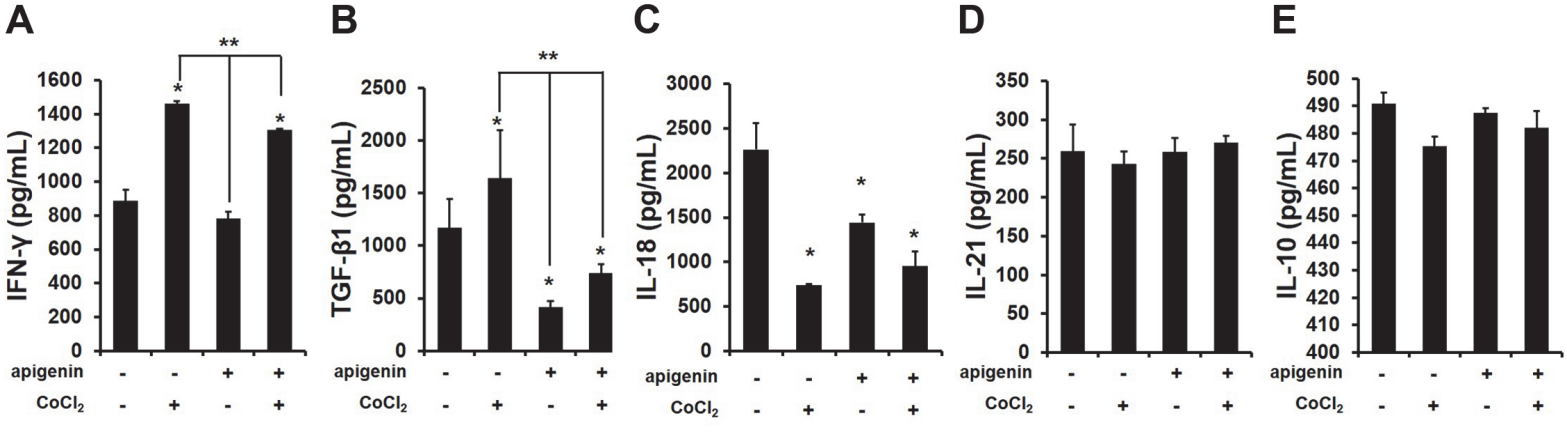
Inhibitory secretion of TGF-β1 from a coculture of NK cells and HCC cells by apigenin. Several cytokines were analyzed in the supernatants of a coculture of NK cells and HCC cells with or without treatment with CoCl_2_ (250 μM) and apigenin (50 μM) using ELISA assay. (**A**) IFN-γ, (**B**) TGF-β1, (**C**) IL-18, (D) IL-21, and (E) IL-10 secreted from a coculture of NK cells and HCC cells. The data are presented as means ± SD from three independent experiments. *p < 0.05 vs non-treated, **p < 0.05 vs CoCl_2_.

Various studies have shown that cytokines in a tumor microenvironment could decide the function of immune cells against cancer cells [23]. Therefore, we examined cytokines secreted from a coculture of NK cells and HCC cells by apigenin treatment. Initially, we identified the level of IFN-γ in the supernatant of a coculture of NK-92 and HCC cells in the presence or absence of apigenin. Unexpectedly, the quantity of IFN-γ in the supernatant of a coculture of NK-92 cells and HCC cells significantly increased when HIF-1α was expressed by treatment with CoCl_2_ (250 μM) as compared with no expression of HIF-1α, and apigenin conversely rather decreased the level of IFN-γ (Fig. 5A; *, **p < 0.05). Furthermore, the expression of HIF-1α in HCC cells cocultured with NK-92 cells significantly increased the level of TGF-β; however, that was remarkably decreased by apigenin (Fig. 5B; *, **p < 0.05). The cytokines IL-18, IL-21 and IL-10, which can be correlated with NK cell activity, were not greatly affected by apigenin, albeit IL-18 decreased (Figs. 5C-5E; *p < 0.05).

## Discussion

In this study, apigenin showed an increase in NK cell cytotoxic effect to HCC cells or HCC cells expressing HIF-1α in vitro. Various cancer types have shown a low frequency and dysfunction of NK cells, and that might promote the metastasis of cancer cells [24]. Moreover, NK cells in patients with HCC commonly present low cytotoxicity and decreased production of interferon (IFN)-γ [11].

Hypoxia is a low-oxygen (O_2_ ≤ 1%) condition generated by cancer metabolism and it induces DNA damage. Hypoxia is known to cause genomic changes that tolerate poor nutrition and a hostile microenvironment in tumor cells, and thus tumors are able to survive [25]. HIF-1α is commonly expressed in many tumors, and this inhibits the expression of Bid and pro-apoptotic Bcl-2-family protein [26] and stimulates the expression of survivin, an apoptosis inhibitor [27]. Moreover, the invasion and metastasis of cancer cells is promoted by hypoxia because it can induce the expression of interleukin (IL)-6, platelet-derived growth factor (PDGF) and transforming growth factor (TGF)-β [28]. In this study, we first tried to examine the NK cell function in HCC cells when HIF-1α was expressed. HIF-1α expression between NK and HCC cells attenuated the NK cytotoxic effect to HCC cells (Fig. 1B), and also reduced the secretion of granzyme B (GrzB) from NK cells into target cells (Fig. 1C). GrzB is a unique serine protease found in the lytic granules of NK cells and T lymphocytes (CTL). NK cells recognize target cells and then secrete GrzB and perforin into the interspace between them. GrzB in the cytoplasm of target cells leads to the cleavage of caspases, which induces their apoptosis [29]. The results indicated that the expression of HIF-1α in HCC cells decreased NK cytotoxicity. In addition, the expression of NK-activating receptors NKG2D, NKp30 and NKp44 on the surface of NK cells significantly decreased with HIF-1α expression (Fig. 2), which was implicated in the decline of an apoptotic effect of NK cells on HCC cells through a decrease in the cleavage of caspase-3, -7, -8, and -9 (Fig. 4). Recently, many studies have focused on the composition of chemokines or cytokines within the tumor microenvironment. The pro-inflammatory cytokines IL-1β, TNF, and IL-6 can induce the transition of EMT in head and neck cancers [30] and anti-inflammatory cytokine IL-10 can lead to tumor cell proliferation through an induction of STAT3 activation in gastric cancer cells [31]. Also, the presence of IL-10, TGF-β, and prostaglandins in the tumor microenvironment play the role of immunosuppressors that inhibit the anti-tumor activities of NK, T and B cells [32-34]. Thus, it is necessary to confirm the level of cytokines, especially that concerned with NK activity. IFN-γ is produced by NK cells by stimulating interleukin(IL)-2, and so we examined the level of IFN-γ produced by a coculture of NK cells and HCC cells. In a coculture of NK cells and HCC expressing HIF-α, the level of IFN-γ was significantly increased (Fig. 5A), contrary to our expectations, while the level of TGF-β1 was substantially increased as well (Fig. 5B). Recently, it has been reported that IFN-γ can lead to EMT transition in pancreatic cells [35] or endometrial cancer cells [36] through the stimulation of MUC4 transcription, which is expressed in an aggressive or metastatic tumor phenotype by the activation of STAT1 [37]. Moreover, several studies showed that IFN-γ signaling enhanced the expression of PD-L1 and induced immune suppression [38]. This evidence fits with our result that an increased level of IFN-γ might be not important in NK cytotoxicity to HCC. However, a high level of TGF-β1 seemed to impair NK activity in HCC (Figs. 1-3). In addition, the level of IL-18 significantly decreased (Fig. 5C), and this was considered to not affect the production of IFN-γ from NK cells but had an inhibitory effect on other factors such as the inflammasome in HCC cells. Our previous study showed that the expression of inflammasome (NLRP3) in HCC could attenuate NK cell cytotoxic ability through the low interaction of NKG2D-MICA/B [39]. This result could mean that a coculture of NK cells and HCC cells might change their environment to induce low NK cell activity by expressing HIF-1α.

The anticancer effect of apigenin is shown in various cancer types containing hepatocellular carcinoma SK-Hep1 and BEL-7402 [40]. According to a recent report, apigenin at a concentration of 25 μg/ml increased NK cell proliferation [20], and the NK immunomodulatory effect of apigenin was recently proved through an induction of cytotoxic granule secretion [41]. Thus, we expected that apigenin could enhance NK cell cytotoxicity to HCC cells. Unexpectedly, in a coculture of NK and HCC cells, apigenin treatment did not increase NK cytotoxicity (Fig. 1) but incurred a cleavage of caspase-3 (Fig. 4), which could be an anticancer effect of apigenin. Several studies have reported that apigenin disrupted tumor angiogenesis through the inhibition of HIF-1 and vascular endothelial growth factor (VEGF) in several human cancers [42]. The expression of HIF-1α in tumor cells under normoxia resists cytotoxic T lymphocytes (CTLs) [43] and induces immune escape of pancreatic cancer cells from NK cells through the shedding of MICA, which interacts with NK-activating receptor (NKG2D) [44]. In our study, apigenin decreased HIF-1α expression in HCC cells (Fig. 4A), and increased NK cytotoxicity and a secretion of GrzB into HCC cells (Figs. 1B and 1C). The inhibitory effect of HIF-1α has been shown in human pancreatic cancer cells S2-013 and CD18 [45], but not in HCC cells yet. This result indicates that apigenin could restore the functions of NK activity through an inhibitory effect of HIF-1α by apigenin. Furthermore, the expression of NK-activating receptors was significantly increased on the surface of NK cells in a coculture of HCC cells expressing HIF-1α (Fig. 2). More interestingly, apigenin significantly increased the expression of CD178 (CD95L) on the surface of NK cells cocultured with HCC cells expressing HIF-1α (Fig. 3C). Several recent studies have shown that induce fast GrzB-mediated cell death in target cells contacting NK cells and subsequently sustain NK cytotoxic effect through death receptor-mediated killing [12]. The CD95L (FasL; CD178) was expressed at the surface of activated T lymphocytes and natural killer cells [21, 22]. This ligand binds their receptor CD95 and subsequently recruits FADD, which is an adaptor protein. Recruiting FADD into CD95 triggers caspase-8 and caspase-10 resulting in apoptosis. Our study showed an activation of caspase-8 in HCC cells expressing HIF-1α cocultured with NK cells in the presence of apigenin (Fig. 4C). In addition, apigenin inhibited excellently the level of TGF-β1 in a coculture of NK cells and HCC cells with or without HIF-1α (Fig. 5B). However, NK activity in HCC was significantly different when HIF-1α was expressed.

Taken together, NK cytotoxic effect on HCC cells was dramatically attenuated by expressing HIF-1α. In particular, the expression of HIF-1α between NK and HCC cells significantly decreased secretion of GrzB from NK cells leading to a decreased killing effect of NK cells toward HCC cells. The attenuation of the NK cell cytotoxic effect on HCC cells expressing HIF-1α, however, could be restored by apigenin. Treatment with apigenin in a coculture of NK and HCC under HIF-1α expression increased the NK cytotoxic effect but had no effect when HIF-1α was not expressed. Interestingly, apigenin increased all expressions of CD178 (CD95L;Fas) on the surface of NK cells in a coculture of HCC cells or HCC cells expressing HIF-1α; however, a difference in the increase in the activation of caspases and GrzB was shown when HIF-1α was expressed. In conclusion, apigenin can be a good compound to increase the NK cytotoxic effect on HCC by inducing high expression of CD95L on the surface of NK cells in a hypoxic condition.
